# Indirect Insulin Resistance Indices and Their Cut-Off Values for the Prediction of Post-Transplantation Diabetes Mellitus in Kidney Transplant Recipients

**DOI:** 10.3390/jcm12237296

**Published:** 2023-11-24

**Authors:** Sara Sokooti, Tamás Szili-Török, Hiddo J. L. Heerspink, Robin P. F. Dullaart, Stephan J. L. Bakker

**Affiliations:** 1 Division of Nephrology, Department of Internal Medicine, University Medical Center Groningen, University of Groningen, 9713 GZ Groningen, The Netherlandss.j.l.bakker@umcg.nl (S.J.L.B.); 2 Department of Clinical Pharmacy and Pharmacology, University Medical Center Groningen, University of Groningen, 9713 GZ Groningen, The Netherlands; 3 Division of Endocrinology, Department of Internal Medicine, University Medical Center Groningen, University of Groningen, 9713 GZ Groningen, The Netherlands; dull.fam@12move.nl

**Keywords:** insulin resistance, post-transplantation diabetes mellitus, HOMA-IR, VAI, LAP, TyG index

## Abstract

Background: Insulin resistance plays an important role in the development of post-transplantation diabetes mellitus (PTDM) in kidney transplant recipients (KTRs). Current methods for the direct determination of insulin resistance are complicated and invasive. Therefore, this study aimed to investigate the relevance of indirect insulin resistance indices in relation to the development of PTDM in KTRs. Methods: We included 472 stable outpatient KTRs without diabetes at baseline from a prospective cohort study. Four indirect insulin resistance indices, namely homeostasis model assessment–insulin resistance (HOMA-IR), visceral adiposity index (VAI), lipid accumulation product (LAP), and triglycerides–glucose (TyG) index, were assessed. We analyzed each measure using the receiver operating characteristic (ROC) curve for PTDM development. The optimal cut-off value for each parameter was determined using the Youden index. Results: After a median of 9.6 years (interquartile range (IQR) 6.6–10.2) of follow-up, 68 (14%) KTRs developed PTDM. In Cox regression analyses, all indirect insulin resistance indices associated with incident PTDM were independent of potential confounders. ROC curve was 0.764 (95% CI, 0.703–0.826) for HOMA-IR, 0.685 (95% CI, 0.615–0.757) for VAI, 0.743 (95% CI, 0.678–0.808) for LAP, and 0.698 (95% CI, 0.629–0.766) for TyG index, with respective optimal cut-off values of 2.47, 4.01, 87.0, and 4.94. Conclusions: Indirect insulin resistance indices can be used to predict incident PTDM in KTRs. In addition to HOMA-IR, insulin-free surrogates of insulin resistance might serve as useful methods to identify KTRs at risk of PTDM, thus obviating the necessity to measure insulin.

## 1. Introduction

Post-transplantation diabetes mellitus (PTDM) is an important long-term complication after kidney transplantation, which is estimated to affect up to 30% of kidney transplant recipients (KTRs) [1,2]. PTDM was associated with an increased risk of other serious comorbidities, including infections, cardiovascular disease, graft failure, and mortality [3]. Similar to the pathogenesis of type 2 diabetes, PTDM may be a result of variable degrees of insulin resistance and impaired insulin secretion by the pancreatic β-cells [4]. Concerning insulin resistance, it has been shown that pre-transplantation insulin resistance in the final stage of kidney failure, obesity, low physical activity, and chronic use of calcineurin inhibitors and corticosteroids are important determinants of insulin resistance in KTRs [5,6,7,8].

Insulin resistance determination in KTRs may be an important step for establishing preventive measures and initiating optimal therapeutic approaches, allowing for early intervention through the early identification of KTRs at increased risk of PTDM. Current gold-standard methods for determining insulin resistance, such as the hyperinsulinemic-euglycemic (HIEG) clamp technique and minimal model approximation of the metabolism of glucose (MMAMG), are expensive, time-consuming, and invasive [9,10,11]. Indirect insulin resistance indices, including homeostasis model assessment–insulin resistance (HOMA-IR), visceral adiposity index (VAI), lipid accumulation product (LAP), or triglycerides-and-glucose (TyG) index, are accepted for epidemiological or clinical studies in the general population because of their simplicity [12,13,14]. Previously, the association between HOMA-IR, the TyG index, and TyG-BMI with PTDM development has been demonstrated in a limited number of studies with small populations of KTRs [15,16,17,18,19]. However, in stable KTRs, it is unknown to what extent these indices could be useful for determining insulin resistance as a predictor of PTDM during a long follow-up time. Moreover, valid cut-off values of the indices have not yet been established for PTDM prediction. Therefore, we aimed to prospectively investigate the association between indirect insulin resistance indices and incident PTDM, as well as their cut-off values for predicting PTDM in KTRs.

## 2. Materials and Methods

### 2.1. Design and Study Population

In this prospective cohort study, all KTRs who survived with a functioning allograft beyond the first year after transplantation between August 2001 and July 2003 were considered eligible to participate. A detailed setup of this prospective longitudinal cohort study has been published elsewhere [20]. Briefly, patients with known systemic illnesses such as heart failure, cancer other than cured skin cancer, or overt generalized infections at baseline or undergoing assessment of insulin resistance were excluded. A total of 606 from an eligible 847 KTRs (72% consent rate) signed written informed consent. At baseline, we excluded 105 recipients from the analysis with pre-existing diabetes (defined as fasting plasma glucose ≥ 7.0 or the use of glucose-lowering/antidiabetic medication) [21]. Additionally, 29 participants with missing insulin values were also excluded, leaving 472 KTRs without diabetes at baseline for the present analysis. The Institutional Review Board approved the study protocol (METc 2001/039). Funding sources had neither a role in the collection and analysis of data nor the publication of the manuscript.

### 2.2. Data Collection

Relevant transplant recipient characteristics such as age, sex, and date of transplantation were extracted from the Groningen Renal Transplant Database. We found the current medication information in the medical record and obtained information on employment status, living situation, smoking and alcohol consumption, and cardiovascular history through a self-report questionnaire.

Blood pressure was recorded as the average of three automated measurements with 1 min intervals after a 6 min rest in the supine position (Omron M4; Omron Europe B.V., Hoofddorp, The Netherlands). BMI was calculated as weight in kilograms divided by height in meters squared. Waist circumference (WC) was measured on bare skin midway between the iliac crest and the 10th rib. Waist-to-height ratio (WHtR) was defined as the waist circumference divided by the body height.

Venous blood was drawn in the morning after an 8–12 h overnight fasting period. Plasma glucose was measured using the glucose oxidase method (YSI 2300 Stat Plus; Yellow Springs Instruments, Yellow Springs, OH, USA). The estimated glomerular filtration rate (eGFR) was calculated through applying the Chronic Kidney Disease Epidemiology Collaboration equation [22]. Serum total cholesterol, triglycerides, and high-density lipoprotein cholesterol (HDL) cholesterol (HDL-C) were measured as described previously [20]. Total cholesterol was determined using the cholesterol oxidase-phenol aminophenazone method (MEGA AU510). LDL cholesterol was calculated using the Friedewald equation. HDL-C was measured using the cholesterol oxidase-phenol aminophenazone method on a Technicon RA-1000 (Bayer Diagnostics, Mijdrecht, The Netherlands). Plasma triglycerides were determined using the glycerol-3-phosphate oxidase-oxidase method (YSI 2300 Stat Plus). Fasting insulin levels at baseline were determined for research purposes using an AxSYM autoanalyzer (Abbott Diagnostics, Abbott Park, IL, USA). HOMA-IR was calculated as (glucose [mmol/L] × insulin [µU/mL])/22.5). VAI was calculated as (waist circumference [cm]/ (39.68 + (1.88 × Body mass index [BMI])) × (triglycerides [mmol/L]/1.03) × (1.31/HDL-C [mmol/L])) for men or (waist circumference [cm]/(39.58 + (1.89 × Body mass index [BMI])) × (triglycerides [mmol/L]/0.81) × (1.51/HDL-C [mmol/L])) for women. LAP was calculated as (waist circumference [cm] − 65) × (triglycerides [mmol/L]) for men or (waist circumference [cm] − 58) × (triglycerides [mmol/L]) for women. TyG index was calculated as Ln (fasting glucose [mg/dL] × triglycerides [mg/dL])/2. TyG-BMI was calculated as TyG × BMI. TyG-WC was calculated as TyG × WC [23], and TyG-WHtR was calculated as TyG × WHtR [24].

### 2.3. Outcome Definition

All measurements at the baseline of our study were taken 6.1 (2.6–11.7) years after transplantation. PTDM was defined according to the Expert Panel recommendations based on the American Diabetes Association criteria [25,26]. The diagnosis was based on the following criteria: classic symptoms (unexplained weight loss, polydipsia, and polyuria), fasting (plasma glucose concentration >126 mg/dL (7.0 mmol/L) after an absence of calory intake for at least 8 h), a non-fasting plasma glucose concentration of >200 mg/dL (11 mmol/L), or the use of glucose-lowering medication (metformin or other glucose-lowering drugs were not used in participants without diabetes at baseline). If glucose was elevated, a confirmatory laboratory test of venous plasma was performed on a subsequent day or at the next visit, after which the diagnosis of PTDM was made.

### 2.4. Statistical Analyses

All statistical analyses were performed using statistical packages SPSS (version 28.0.1; IBM, Armonk, NY, USA) and Stata/SE (version 14; StataCorp, College Station, TX, USA). Two-sided *p*-values ˂ 0.05 were considered as statistically significant. A sample size of 366 participants provided 90% power to detect a hazard ratio for the association between HOMA-IR and post-transplantation diabetes of 1.73, assuming a type 1 error of 0.05 [15]. All the variables were checked for normality. Data with skewed distribution were expressed as median (interquartile range), and data with normal distribution were expressed as mean ± SD. Absolute numbers (percentages) were given for categorical variables. For the variables with a skewed distribution, logarithmic transformation was used to approximate normality. Univariate linear regression analyses were performed to assess the cross-sectional associations between insulin resistance indices.

To determine if indirect insulin resistance indices were prospectively associated with the risk of PTDM, crude and multivariable Cox proportional hazards regression analyses were performed. In addition, hazard ratios (HR) with 95% confidence intervals (CIs) were calculated per 1 SD increment of predictors (loge transformed for variables that were not normally distributed). The proportional hazards assumption was tested for each predictor along with covariates to see if it was violated. All models were adjusted for the time since transplantation, systolic blood pressure (SBP), eGFR, and medications (prednisolone dosage, calcineurin inhibitors, and proliferation inhibitors). Subsequently, we performed additive adjustments in Cox regression analyses to avoid the inclusion of too many or similar covariates.

We analyzed each of the indirect insulin resistance indices using the receiver operating characteristic (ROC) curve to estimate the predictive ability for the development of PTDM. The cut-off value of each surrogate measure was determined as the value with the highest Youden index score [27]. Using the cut-off value of each surrogate, multivariable Cox proportional hazards regression models were constructed to evaluate the HR and 95% CI for PTDM development as a dichotomous variable.

For the validation of our models, we performed multivariable Cox proportional hazards regression analyses adjusted for age, sex, family history of diabetes, and BMI [15]. Additionally, to validate the cut-off value of HOMA-IR, we performed multivariable Cox proportional hazards regression models, using HOMA-IR as a categorical variable (HOMA-IR < 2.67 vs. ≥2.67 (μU/mL/12)/22.5) [17].

## 3. Results

### 3.1. Baseline Characteristics

A total of 472 KTRs (56.4% men, 50.4 ± 12.1 years) were included in the study. The time between renal transplantation and the baseline evaluation was 6.1 (2.6–11.7) years. Table 1 shows the further characteristics of KTRs at baseline. The distributions of indirect insulin resistance indices are presented in Table S1.

### 3.2. Cross-Sectional Associations

The correlations between loge-transformed indirect insulin resistance indices are shown in Figure S1. In univariable regression analyses, we found that HOMA-IR, VAI, the TyG index, and LAP were all correlated with each other. The correlation between the TyG index and VAI was the strongest correlation among all indices (standardized regression coefficient β = 0.872).

### 3.3. Prospective Analyses of Insulin Resistance Index and Incident PTDM in KTRs

After a median (interquartile range) follow-up period of 9.6 (6.6–10.2) years, 68 incident cases of PTDM were ascertained. The associations between indirect insulin resistance indices and incident PTDM in KTRs are shown in Table 2. Higher HOMA-IR, VAI, LAP, and the TyG index were all associated with a higher hazard of incident PTDM in crude analyses. The association remained significant after adjustment for age, sex, smoking, time since transplantation, SBP, eGFR, and medication in model 1. Subsequently, we performed additional adjustments for other variables, including BMI, HbA1c, triglycerides, HDL-C, and VAI for HOMA-IR; glucose, HbA1c, and HOMA-IR for VAI; glucose, HbA1c, HDL-C, and HOMA-IR for LAP; and BMI, HbA1c, and HDL-C for the TyG index. The associations remained significant after further adjustment for those variables in all the indirect insulin resistance indices (Table 2).

### 3.4. ROC Curves and Cut-Off Values of Indirect Insulin Resistance Indices for Incident PTDM

**Figure 1:**
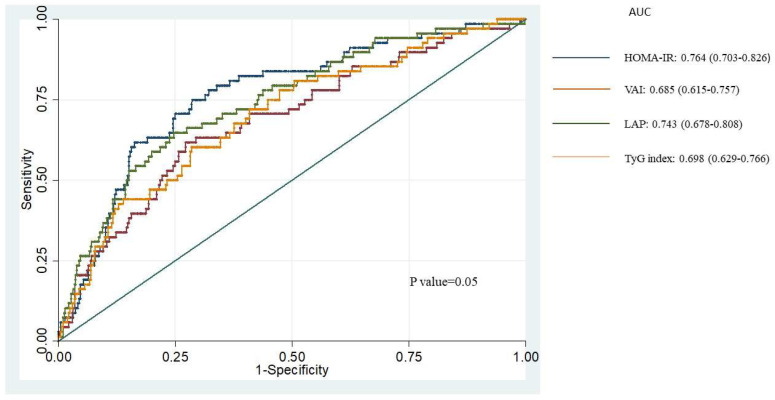
ROC curves of incident PTDM in 9.6 (6.6–10.2) years based on each indirect insulin resistance index. HOMA-IR: homeostasis model assessment–insulin resistance; VAI: visceral adiposity index; LAP: lipid accumulation product; TyG index: triglycerides-and-glucose index.

The ROC for incident PTDM in KTRs in 9.6 (6.6–10.2) years, according to the indices, is presented in Figure 1. The area under the curve (AUC) was 0.764 (95% CI, 0.703–0.826) for HOMA-IR, 0.685 (95% CI, 0.615–0.757) for VAI, 0.743 (95% CI, 0.678–0.808) for LAP, and 0.698 (95% CI, 0.629–0.766) for the TyG index without a significant difference among them (*p* = 0.0517).

The cut-off values for each insulin resistance based on the Youden indices, along with their corresponding sensitivity and specificity for each index, are presented in Table 3. To test this cut-off value, we examined the association between each index and incident PTDM, using each index as a categorical variable (HOMA-IR < 2.47 vs. ≥2.47; VAI < 4.01 vs. ≥4.01; LAP < 87.04 vs. ≥87.04; TyG index < 4.94 vs. ≥4.94). Indirect insulin resistance indices as a categorical variable predicted incident PTDM in the model adjusted for age, sex, smoking, time since transplantation, SBP, eGFR, and medication (Table 3).

### 3.5. Cut-Off Values of Indirect Insulin Resistance Indices Combined with TyG Index for Incident PTDM

Finally, we conducted ROC curve analyses to estimate the predictive value of TyG-BMI, TyG-WC, and TyG-WHtR for the development of PTDM during the follow-up (Table S2). AUC was 0.767 (95% CI, 0.706–0.830) for TyG-BMI, 0.748 (95% CI, 0.681–0.814) for TyG-WC, and 0.711 (95% CI, 0.642–0.780) for TyG-WHtR. We determined the cut-off values along with their corresponding sensitivity and specificity for each index. Subsequently, we examined the association between each index and incident PTDM, using each index as a categorical variable (TyG-BMI < 97.7 vs. ≥97.7; TyG-WC < 389.0 vs. ≥389.0; TyG-WHtR < 5.25 vs. ≥5.25). Indirect insulin resistance indices combined with the TyG index as a categorical variable predicted incident PTDM in the model adjusted for age, sex, smoking, time since transplantation, SBP, eGFR, and medication (Table S2).

### 3.6. Validation of Cox-Regression Model and Cut-Off Value of HOMA-IR

The associations between indirect insulin resistance indices and incident PTDM in KTRs are shown in Table S3 using the model adjusted for age, sex, family history of diabetes, and BMI [15]. Higher HOMA-IR, VAI, LAP, and the TyG index were all associated with a higher hazard of incident PTDM in the new model.

To validate the cut-off value of HOMA-IR, we examined the association between HOMA-IR as a categorical variable (HOMA-IR < 2.67 vs. ≥2.67) and incident PTDM [17]. HOMA-IR, as a categorical variable, predicted incident PTDM after adjustment for confounders, including age, sex, smoking, time since transplantation, SBP, eGFR, medication, and family history of diabetes (Table S4).

## 4. Discussion

In this study, we found that all indirect insulin resistance indices, including HOM-IR, VAI, LAP, and the TyG index, were independently associated with incident PTDM in KTRs. Our study emphasizes the applicability of both HOMA-IR and insulin-free indirect insulin resistance indices in PTDM prediction. Moreover, we investigated and proposed cut-off values of indirect insulin resistance indices for incident PTDM in KTRs.

Insulin resistance is a well-established pathophysiological phenomenon that occurs earlier before elevation in fasting blood glucose in the course of progression from normoglycemia to diabetes [28]. HOMA-IR is a widely used and validated surrogate index estimate of insulin resistance in various populations as well as in pregnant women and in KTRs [11,29,30]. In KTRs, lipid factors, including HDL-C, total cholesterol, remnant cholesterol, and triglycerides, were associated with HOMA-IR and predicted PTDM [31,32]. Obesity, waist circumference, and prednisolone treatment were the main factors associated with insulin resistance following transplantation [33]. Similarly, we found correlations between HOMA-IR and the VAI, LAP, and TyG indices, all of which represent insulin-free metabolic determinants of insulin resistance.

Since insulin is recognized as a marker of insulin resistance, quantifying insulin in humans is of great importance for epidemiological studies and clinical practice [34]. However, no standard assay for measuring fasting insulin has been developed and widely adopted in clinical settings [35]. There are currently both direct and indirect methods employed for these purposes, with varying degrees of complexity [36,37]. Direct methods such as the HIEG clamp technique, MMAMG, and the insulin suppression test directly assess insulin-mediated glucose utilization under steady-state conditions that are both more invasive and time-consuming [9,38]. Accordingly, to avoid using methods for determining insulin resistance, insulin-free surrogate indices for estimating insulin resistance have been investigated in the general population. The VAI, LAP, and TyG indices have been shown to be accurate predictors of insulin resistance and diabetes development in the general population and individuals of various ethnicities [13,14,39,40].

The ability of these indirect insulin resistance indices to predict PTDM has not yet been compared in the setting of metabolically stable KTRs, i.e., at least one year after transplantation. Several studies have shown an association between HOMA-IR and the development of PTDM during the first year after transplantation in various ethnicities [15,16,17]. Additionally, pre-transplantation HOMA-IR, as an insulin resistance marker, has been linked to the development of PTDM [18,41]. Recently, Xiaojie et al. found that the TyG index and TyG BMI are associated with the development of PTDM within 6 months after kidney transplantation [19]. In alignment with prior studies, our investigation delves into the association between these indirect insulin resistance indices and PTDM development in a larger population of kidney transplant recipients (KTRs) with an extended follow-up period. Our study exclusively included KTRs with a functioning graft of more than one year after transplantation, thus limiting the impact of transient post-transplantation hyperglycemia. This exclusionary approach aimed to prevent the overestimation of PTDM incidents [25]. Transient post-transplantation hyperglycemia typically resolves when the dosage of immunosuppressive drugs is tapered, which was taken into account in our study by including stable KTRs [42].

Chakkera et al. developed a pretransplant risk score for the prediction of PTDM that took into account factors such as recipient age, corticosteroid therapy following transplantation, gout medication prescription, BMI, fasting glucose and triglycerides, and family history of type 2 diabetes [43]. The AUC with different variable constructions ranged from 0.70 to 0.72. In the current study, the AUC was 0.764 for HOMA-IR, 0.685 for VAI, 0.743 for LAP, and 0.698 for the TyG index. Although the HOMA-IR and LAP indices had a higher AUC compared with the VAI and TyG indices, the differences were not statistically significant, possibly due to the lack of power. Furthermore, we found that the AUC of the TyG index, when combined with BMI, WC, and waist-to-height ratio, was comparable to the AUC of the HOMA-IR. It is consistent with previous studies that show the diagnostic value of insulin resistance in predicting metabolic syndrome development [44,45]. The results of this study suggest that indirect insulin resistance indices can identify KTRs at increased risk of PTDM.

In KTRs, no valid cut-off value for the clinical use of HOMA-IR, VAI, LAP, and the TyG index has been determined. Several studies used the percentile criterion to determine HOMA-IR cut-off values in the general population [46,47,48]. Based on the 75th percentile of HOMA-IR, those cut-off values varied greatly according to the participant characteristics and were estimated to be 2.53 mU/L2 in Koreans, 1.6 mU/L2 in Iranians, 2.0 mU/L2 in Swedish men, and 3.8 mU/L2 in French men [35,46,47,48]. Recently, HOMA-IR cut-off values were determined using the value with the highest Youden index score for predicting the development of diabetes in different populations. For instance, the corresponding numbers for Iranian men were 2.17, Iranian women were 1.8, Southern Chinese were 1.97, and Koreans were 1.83 [39,49,50]. The TyG index cut-off value for predicting incident diabetes is predicted to be 4.68 in healthy Mexicans [13]. In order to forecast the development of diabetes in healthy Koreans, Kim et al. evaluated the cut-off values of indirect insulin resistance indices, which were 2.54 for VAI, 36.6 for LAP, and 4.69 for the TyG index [39]. Dedinska L et al. found that the HOMA-IR cut-off value for predicting PTDM was 2.67 (μU/mL/12)/22.5, which aligns with the results of our study [17]. Interestingly, when compared with previous studies, the results obtained in our study showed approximately two times higher cut-off values for these indirect insulin resistance indices among KTRs. This seems likely because KTRs are likely to be more insulin resistant due to medication and metabolic factors after transplantation than non-diabetic subjects from the general population [51]. Recently, Xiaojie et al. found the TyG index and TyG-BMI cut-off values for predicting PTDM in Chinese KTRs [19]. Those cut-off values were approximately two-fold higher (9.06 and 198.09, respectively) compared with our study. This discrepancy could be attributed to the fact that their indices were measured within three months after transplantation, and a diagnosis of PTDM was made within six months after transplantation. This difference in timing may necessitate accounting for the effects of immunosuppressive medication and transient post-transplantation hyperglycemia.

Notably, HOMA-IR was not significantly better at identifying KTRs at higher risk of PTDM than other indirect insulin resistance indices. This may indicate that insulin-free equations could be used as a simple and reliable method to identify KTRs at increased risk of PTDM as well.

Our study had a prospective design conducted in a relatively large population of stable KTRs. Furthermore, despite 9.6 years of follow-up, all patients had complete endpoint evaluations.

## 5. Study Limitations

This study had certain limitations. First, it was conducted at a single center, and most of the participants were White. However, other studies conducted in different ethnicities have confirmed the association between HOMA-IR, the TyG index, and TyG-BMI with the development of PTDM during the first year after transplantation. This suggests the necessity to validate our findings in other ethnicities beyond one year post transplantation. The second limitation is that oral glucose tolerance tests were not carried out in our cohort study, which could result in an underestimation of PTDM frequency. Of note, however, we applied the American Diabetes Association criteria to diagnose PTDM.

## 6. Conclusions

In conclusion, besides HOMA-IR, insulin-free surrogates of insulin resistance, in particular LAP and the TyG index, could serve as useful methods to identify KTRs at risk of PTDM development, thus obviating the necessity to measure insulin. The cut-off values proposed here might be useful to apply indirect insulin resistance indices to predict incident PTDM in KTRs.

## Figures and Tables

**Table 1:** Baseline characteristics of KTRs according to incident PTDM.

Variables	
Participants, *n*	472
General characteristics	
Men, %	56.4
Age, year	50.4 ± 12.1
Current smoker, %	40.9
Alcohol use, never, %	45.1
Weight, kg	76.3 ± 13.5
Height, cm	172.5 ± 9.7
BMI, kg/m^2^	25.6 ± 4.1
Transplant demographics	
Time since renal transplantation, year	6.1 (2.6–11.7)
Donor age, year	39.5 (23.0–51.0)
Living donor, %	15.0
Dialysis duration, months	27 (13–49)
Rejection, %	45.5
Blood pressure	
Systolic blood pressure, mmHg	151.4 ± 22.4
Diastolic blood pressure, mmHg	898.8 ± 9.9
Metabolic variables	
Total cholesterol, mmol/L	5.6 (5.0–6.2)
LDL cholesterol, mmol/L	3.6 (3.0–4.1)
HDL cholesterol, mmol/L	1.1 (0.9–1.3)
Triglycerides, mmol/L	1.9 (1.4–2.6)
Glucose Homeostasis	
Glucose, mmol/L	4.5 ± 0.5
HbA1c, %	6.3 ± 0.8
Insulin, μU/mL	10.3 (7.7–14.1)
hs-CRP, mg/L	1.9 (0.7–4.6)
Renal function	
Serum Creatinin mmol/L	137.0 (113.0–171.0)
eGFR, mL/min per 1.73 m^2^	46.7 (35.7–57.6)
UAE, g/24 h	0.2 (0.0–0.50)
CMV infection, %	29.2
Medication use	
Statin use, %	47.9
Anti-hypertensive medication, %	85.4
Prednisolone, mg/day	9.2 ± 1.3
Calcineurin inhibitor, %	77.3
Cyclosporine, %	63.8
Tacrolimus, %	13.6
Proliferation inhibitor, %	55.8
Azathioprine,%	33.9
Mycophenolic acid, %	41.9

Continuous variables are reported as mean ± SD or median (interquartile range), and categorical variables are reported as percentages. BMI: body mass index; HDL: high-density lipoprotein; HOMA-hs-CRP: high-sensitivity C-reactive protein; CMV: cytomegalovirus; eGFR: estimated glomerular filtration rate; UAE: urinary albumin excretion.

**Table 2:** Association between indirect insulin resistance indices and risk of PTDM in 472 KTRs.

HOMA-IR, *n* = 68	HR (95% CI) Per 1 SD	*p* Value
Crude	2.61 (1.99–3.42)	<0.001
Model 1+	2.79 (2.07–3.76)	<0.001
+BMI	2.26 (1.62–3.14)	<0.001
+HbA1C	2.37 (1.75–3.22)	<0.001
+TG, HDL-C	2.35 (1.71–3.22)	<0.001
+VAI	2.30 (1.68–3.16)	<0.001
VAI, *n* = 68	HR (95% CI) Per 1 SD	*p* Value
Crude	1.97 (1.53–2.53)	<0.001
Model 1+	2.09 (1.62–2.70)	<0.001
+Glucose	1.92 (1.49–2.47)	<0.001
+HbA1c	2.12 (1.61–2.79)	<0.001
+HOMA-IR	1.57 (1.20–2.04)	0.001
LAP, *n* = 68	HR (95% CI) Per 1 SD	*p* Value
Crude	2.39 (1.84–3.10)	<0.001
Model 1+	2.62 (1.98–3.46)	<0.001
+glucose	2.40 (1.82–3.16)	<0.001
+HbA1c	2.67 (1.99–3.57)	<0.001
+HDL-C	2.62 (1.95–3.51)	<0.001
+HOMA-IR	1.94 (1.44–2.63)	<0.001
TyG index, *n* = 68	HR (95% CI) Per 1 SD	*p* Value
Crude	2.38 (1.83–3.10)	<0.001
Model 1	2.58 (1.96–3.39)	<0.001
+BMI	2.10 (1.57–2.83)	<0.001
+HbA1C	2.48 (1.85–3.31)	<0.001
+HDL-C	2.52 (1.89–3.36)	<0.001

HRs (95% CIs) are given per 1 SD increase and were derived from Cox proportional hazard analyses adjusted for age, sex, smoking, time since transplantation, SBP, eGFR, medications (prednisolone dosage, calcineurin inhibitors, proliferation inhibitor) in model 1. HOMA-IR: homeostasis model assessment–insulin resistance; TyG index: triglycerides-and-glucose index; HR: hazard ratio; SBP: systolic blood pressure.

**Table 3:** Cut-off values indirect insulin resistance indices with their corresponding sensitivity, specificity, and hazard ratio (HR).

Insulin Resistance Index	Cut-Off Value	Sensitivity	Specificity	HR (95% CI)
HOMA-IR	2.47	75.0	71.3	6.42 (3.67–11.25)
VAI	4.01	61.8	72.8	4.10 (2.48–6.77)
LAP	87.04	64.7	75.2	5.82 (3.46–9.80)
TyG index	4.94	51.5	83.9	5.73 (3.45–9.52)

HRs (95% CIs) were derived from the Cox proportional hazard model adjusted for age, sex, smoking, time since transplantation, SBP, eGFR, and medications (prednisolone dosage, calcineurin inhibitors, and proliferation inhibitor). HOMA-IR: homeostasis model assessment–insulin resistance; TyG index: triglycerides-and-glucose index; HR: hazard ratio; SBP: systolic blood pressure.

## Data Availability

The data presented in this study are available on request from the corresponding author.

## Supplementary Materials

The following supporting information can be downloaded at https://www.mdpi.com/article/10.3390/jcm12237296/s1, Table S1: Distributions of the indirect insulin resistance indices at baseline examination; Figure S1: Univariate analyses of indirect insulin resistance indices with each other; Table S2: AUC and cut-off values of indirect insulin resistance indices combined with the TyG index with their corresponding sensitivity, specificity, and hazard ratio (HR). Table S3: Association between indirect insulin resistance indices and risk of PTDM in 472 KTRs. Table S4: The association between HOMA-IR as a categorical variable (HOMA-IR < 2.67 vs ≥2.67) and incident PTDM.
