# Supplementary material for: Indirect Insulin Resistance Indices and Their Cut-Off Values for the Prediction of Post-Transplantation Diabetes Mellitus in Kidney Transplant Recipients
Source: J Clin Med. 2023 Nov 24;12(23):7296. doi: 10.3390/jcm12237296 (PMC10707270; doi:10.3390/jcm12237296)
Supplement: Supplementary file 1 [file jcm-12-07296-s001.zip › jcm-2700509-supplementary.pdf]

Table S1. Distributions of the indirect insulin resistance indices at baseline examination

		Percentile			
Insulin resistance index	10th	25th	50th	75th	90th
HOMA-IR, mU/L^2	1.03	1.49	2.03	2.90	4.05
VAI	1.25	1.87	2.82	4.47	6.33
LAP	23.59	36.31	58.74	94.78	141.19
TyG index	4.42	4.59	4.74	4.92	5.0
TyG-BMI	96.95	106.90	119.10	135.64	149.89
TyG-WC	364.25	398.24	451.75	499.08	554.98
TyG-WHtR	3.82	4.16	4.57	4.99	5.41

HOMA-IR: homeostasis model assessment-insulin resistance; VAI: visceral adiposity index; LAP: lipid accumulation product; TyG index: triglycerides and glucose index

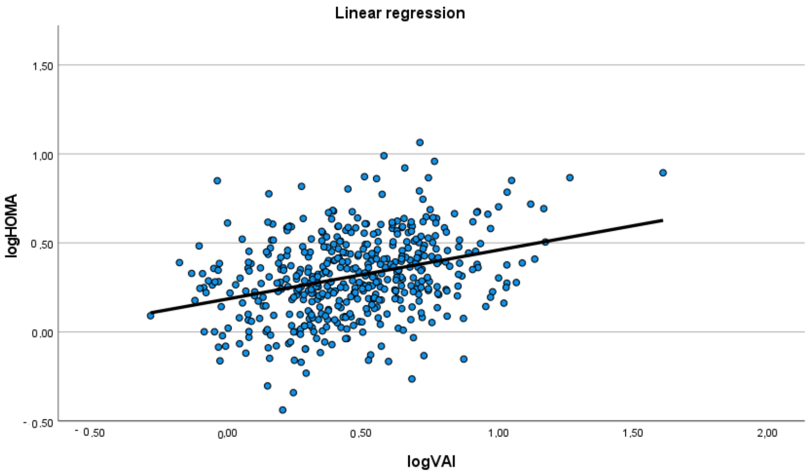

Standardized coefficient β= 0.324 (0.200-0.344) p <0.001

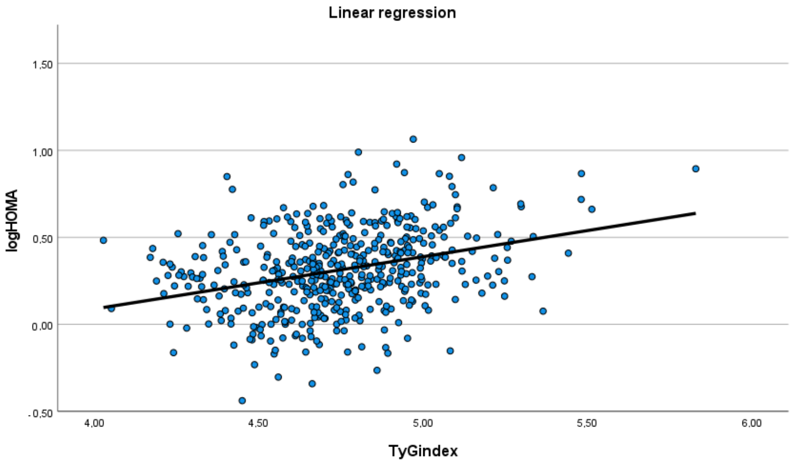

Standardized coefficient β= 0.325 (0.221-0.380) p <0.001

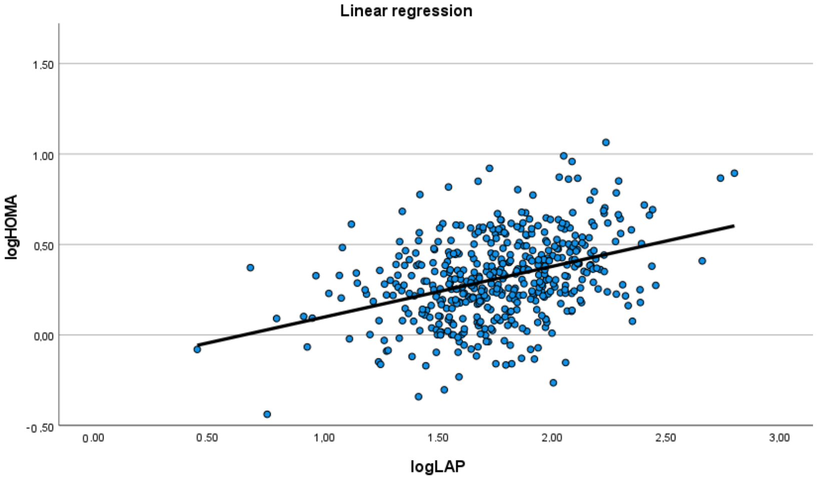

Standardized coefficient β= 0.383 (0.218-0.341) p <0.001

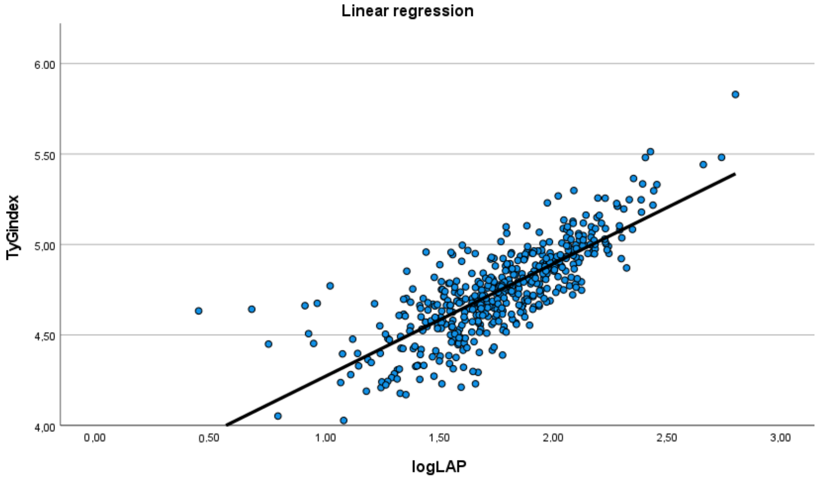

Standardized coefficient β= 0.786 (0.577-0.665) p <0.001

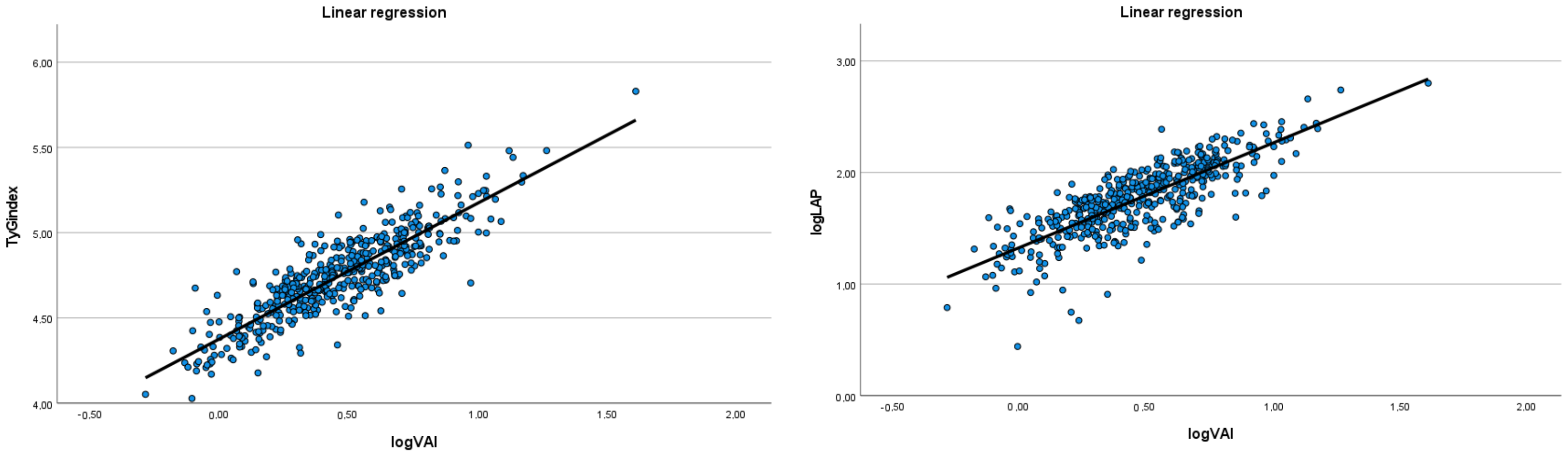

Standardized coefficient β= 0.872 (0.752-0.833) p <0.001

Standardized coefficient β= 0.812 (0.660-0.753) p <0.001

Figure S1. Univariate analyses of indirect insulin resistance indices with each other. β are reported as Standardized coefficient β.
HOMA-IR: homeostasis model assessment-insulin resistance; VAI: visceral adiposity index; LAP: lipid accumulation product; TyG index: triglycerides and glucose index

Table S2. AUC and cut-off values indirect insulin resistance indices combined with TyG index with their corresponding sensitivity, specificity, and hazard ratio (HR).

Insulin resistance index combined with TyG index	AUC (95% CI)	Cut-off value	Sensitivity	Specificity	HR (95% CI)
TyG-BMI	0.767 (0.706-0.830)	97.7	67.7	75.2	5.24 (1.26-21.82)
TyG-WC	0.748 (0.681-0.814)	389.0	61.8	80.7	4.27 (1.67-10.94)
TyG-WHtR	0.711 (0.642-0.780)	5.25	60.3	79.0	4.32 (2.53-7.38)

HRs (95% CIs) were derived from Cox proportional hazard model adjusted for age, sex, smoking, time since transplantation, SBP, eGFR, medication use (prednisolone dosage, calcineurin inhibitors, proliferation inhibitor).

AUC: area under the curve; HOMA-IR: homeostasis model assessment-insulin resistance; VAI: visceral adiposity index; LAP: lipid accumulation product ; TyG index; triglycerides-glucose index; HR: hazard ratio; SBP: systolic blood pressure

Table S3. Association between indirect insulin resistance indices and risk of PTDM in 472 KTRs

Indirect insulin resistance indices	**HR (95% CI) Per 1 SD**	**p Value**
HOMA-IR *	2.06 (1.52-2.80)	<0.001
VAI	1.99 (1.55-2.55)	<0.001
LAP	2.46 (1.90-3.19)	<0.001
TyG index *	1.87 (1.41-2.48)	<0.001

HRs (95% CIs) were derived from Cox proportional hazard analyses adjusted for age, sex, and family history of diabetes. * Additionally adjusted for BMI
HOMA-IR: homeostasis model assessment-insulin resistance; TyG index; triglycerides and glucose index; HR: hazard ratio.

Table S4. the association between HOMA-IR as a categorical variable (HOMA-IR<2.67 vs ≥2.67) and incident PTDM

	**HR (95% CI)**	**p values**
HOMA-IR as categorical variable		
Model 1	4.89 (2.95-8.09)	<0.001
Model 2	4.95 (2.99-8.20)	<0.001

HRs (95% CIs) were derived from Cox proportional hazard analyses adjusted for age, sex, and BM Iin model 1 and Additionally adjusted for family history of diabetes in model 2.
